# Molecular Identification and Antimicrobial Potential of *Streptomyces* Species from Nepalese Soil

**DOI:** 10.1155/2020/8817467

**Published:** 2020-08-27

**Authors:** Karan Khadayat, Dawa Dindu Sherpa, Krishna Prakash Malla, Sunil Shrestha, Nabin Rana, Bishnu P. Marasini, Santosh Khanal, Binod Rayamajhee, Bibek Raj Bhattarai, Niranjan Parajuli

**Affiliations:** ^1^Central Department of Chemistry, Tribhuvan University, Kirtipur, Kathmandu, Nepal; ^2^Department of Biotechnology, National College, Tribhuvan University, Naya Bazar, Kathmandu, Nepal; ^3^Department of Microbiology, National College, Tribhuvan University, Naya Bazar, Kathmandu, Nepal

## Abstract

*Streptomyces* are widely used for the production of secondary metabolites with diverse biological activities, including antibiotics. The necessity of alternative antimicrobial agents against multidrug-resistant pathogens is indispensable. However, the production of new therapeutics is delayed in recent days. Thus, the isolation of new *Streptomyces* species has drawn attention. Nepal—a country rich in biodiversity—has got high possibilities for the discovery of members of actinomycetes, especially in the higher altitudes. However, in vain, only a few screening research works have been reported from Nepal to date. *Streptomyces* species were isolated on ISP4 media, and characterization was performed according to morphological similarity and 16S rRNA sequence similarity using bioinformatic tools. Ethyl acetate extracts of *Streptomyces* species were prepared, and the antimicrobial activity was carried out using agar well diffusion technique. In this report, 18 *Streptomyces* species isolated from the soil were reported based on sequence analysis of 16S rRNA. Among them, 12 isolates have shown antibacterial activity against extended-spectrum beta-lactamase- (ESBL-) producing *Escherichia coli.* Here, we have also analyzed 16S rRNA in 27 *Streptomyces* species whose whole-genome sequence is available, which has revealed that some species have multiple copies of the 16S gene (∼1.5 kb) with significant variation in nucleotides. In contrast, some *Streptomyces* species shared identical DNA sequences in multiple copies of 16S rRNA. The sequencing of numerous copies of 16S rRNA is not necessary, and the molecular sequencing of this region is not sufficient for the identification of bacterial species. The *Streptomyces* species-derived ethyl acetate extracts from Nepalese soil demonstrate potential activity against ESBL-producing *E. coli.* Thus, they are potential candidates for antibiotics manufacturing in the future.

## 1. Background

Multidrug-resistant pathogens have drawn global attention as a significant challenge to treat and prevent the growing number of infectious diseases [1, 2]. Such resistance typically occurs as a result of drug inactivation, target alteration, and reduced accumulation unsettled to decreased permeability and/or increased efflux [3]. Novel organisms with the capacity to produce new secondary metabolites or therapeutic agents are immediately required to combat the fatal diseases caused by such antibiotic-resistant pathogens [4, 5]. Researchers have continuously explored novel, sustainable, potent, and broad-spectrum bioactive compounds from diverse sources, including *Streptomyces* species [6]. *Streptomyces*, the largest genus of class actinobacteria, is a Gram-positive, spore-forming, filamentous, and aerobic bacterium [7]. *Streptomyces* genome contains more than 20 gene clusters to secondary metabolites of higher clinical importance, including antibiotics that could tackle the rise of antimicrobial resistance [8]. About two-thirds of natural antibiotics are isolated from actinomycetes, of which 70% is produced from *Streptomyces* and accounts for 75% of clinically available antibiotics used in the treatment of multiple infections in humans [9–12]. Many studies report that *Streptomyces* have tremendous potential to yield secondary metabolites, including anticancer drugs, antibiotics, growth factors, and herbicides [13]. The higher percentage of GC content (∼70%) is present in *Streptomyces* spp. The 16S rDNA analyses and DNA-DNA hybridization are significant distinguishing properties that separate *Streptomyces* from other actinobacteria. *Streptomyces* from extreme or untouched habitats such as high altitude [14, 15] and desert [16, 17] are isolated and cultured to discover novel antibiotics. Extended-spectrum beta-lactamases (ESBLs) produced primarily by the bacteria *Escherichia coli* and *Klebsiella pneumoniae* confer resistance to most beta-lactam antibiotics, including penicillins and cephalosporins. Infections caused by these bacteria are being difficult to treat globally, and the mortality rate has sharply increased along with prolonged hospital stay and greater economic burden [18].

Due to the unique geographical niche of Nepal, soil microbes, such as *Streptomyces,* have a high probability of producing novel secondary metabolites of diverse clinical value, which is much anticipated in the health care sector. In this report, soil samples from 14 different environments of Nepal with varying from 86 m to 4,026 m above the sea level in altitude were collected for the isolation of *Streptomyces* species with antibacterial properties. This study is mainly aimed at the screening of *Streptomyces* species that can produce metabolites for the inhibition of ESBL-producing clinical isolate of *E. coli,* and their molecular identification using 16S rRNA sequencing. Besides, 16S rRNA found in other *Streptomyces* species reported in GenBank is also reviewed for further scientific discourse.

## 2. Methods

### 2.1. Collection and Pretreatment of Soil Sample

The soil samples were collected from 14 different habitats of Nepal (altitude from 86 m to 4,026 m above the sea level) such as gardens, cultivated fields, barren land, open fields, organic manure pits, and soil from conserved forests, as shown in Supplementary Materials (Table S1). The samples were collected digging around 5–10 cm depth from the surface of the earth. Soil samples were then packed in sterile polythene bags, labeled, and brought to the laboratory. They were air-dried for 3-4 hrs at 45°C, crushed, and sieved before use for the isolation of *Streptomyce*s species [19].

### 2.2. Isolation of *Streptomyces*

One gram of soil was dissolved in 10 mL of sterile distilled water in the test tube and vortexed vigorously for 5 minutes. Then, the suspension was heated at 80°C for 30 minutes and serially diluted up to 10^−4^ in the laminar hood; then 100 *µ*L of each dilution was placed on the International *Streptomyces* Project 4 (ISP4) agar medium supplemented with nalidixic acid (20 mg/mL) and cycloheximide (50 mg/mL) (ISP4: 10 gm starch, 1 gm dipotassium phosphate (K_2_HPO_4_), 1 gm calcium carbonate, 1 mg ferrous sulfate, 1 mg manganese chloride, 1 mg zinc sulfate, 18 gm Bacto agar, 1 gm sodium chloride, 2 gm ammonium sulfate, and 1000 mL distilled water at pH 7.0 ± 0.1). The inoculum was then appropriately spread using a sterile glass spreader until the plates were dry and incubated at 28°C for 7–12 days [20].

### 2.3. Characterization of *Streptomyces*

The characterization of *Streptomyces* was performed based on their Gram staining, growth pattern, colony morphology, and the formation of soluble pigments, as suggested by Bergey's Manual of Systematic Bacteriology, Second Edition, Vol. 5, The Actinobacteria, Part A [21]. All of the isolates were grown on ISP4 media, and the morphology of each isolate was visually observed (such as colony characteristics, an earthy odor, spore formation, and aerial and substrate mycelia of colonies). Sugar utilization tests and physiological tests such as motility, salt tolerance, and temperature tolerance were also performed.

### 2.4. Genomic DNA Extraction and 16S rRNA Amplification of Isolated *Streptomyces* Strains

Phenotypically identified isolates of *Streptomyces* were cultured on trypticase soy broth (TSB) using glass beads in 100 ml conical flasks for 3 days at 28°C. After incubation, the supernatant part was discarded, and mycelia were harvested from the broth by centrifugation at 4,000 rpm for 15 min. Genomic DNA was isolated by the standard phenol–chloroform method from the harvested mycelia [22]. The universal primers 27 F:5′-AGAGTTTGATCMTGGCTCAG-3′ and 1492 R:5′-ACGGYTACCTTGTTACGACTT-3′ were used for amplification of 16S rRNA from genomic DNA. The amplification was carried out in a 50 *μ*L of total volume by using 125 ng of genomic DNA as a template with 2X premix (*Taq* polymerase) and 10 *μ*M of each primer. PCR conditions were maintained as follows: initial 5 min for denaturation at 95°C, followed by 35 cycles of 30s at 95°C, 30s at 51.4°C, and 120s at 72°C, and a final extension of 10 min at 72°C (TAKARA Thermal Cycler, Japan). The amplified products were examined by 0.8% agarose gel electrophoresis [23] stained by ethidium bromide. The purified PCR products were sequenced using the same primers, 27 F and 1492R (Macrogen, Inc., South Korea).

### 2.5. Sequence Analysis

The homology search of partial DNA sequences of 16S rRNA was performed by comparing them with the public database (NCBI) using the standard basic local alignment search tool (BLAST) program. Multiple alignments were conducted using Clustal [24], and the phylogenetic tree was made using MEGA version 6.0 by the neighbor-joining method with bootstrap values calculated from 1,000 replications (Supplementary Materials, Figure S1) [25]. On the other hand, the multiple copies of 16S rRNA present in the assembled genome sequencing of the other 27 *Streptomyces* species given in the website (JGI IMG Integrated Microbial Genomes and Microbiomes, 2019) was evaluated through multiple DNA-DNA sequence alignments using Clustal W (Supplementary Materials, Table S3).

### 2.6. Extraction of Bioactive Compounds

The isolated *Streptomyces* species were first cultured in the TSB and incubated at 30°C for three days for the preparation of inoculum. After full growth, 1% *Streptomyces* mycelium was transferred aseptically into the fresh TSB and again incubated at 30°C for seven days. An equal volume of organic solvent, ethyl acetate, was used for extracting the bioactive compounds to an equal volume of culture. The mixture of culture with an organic solvent was shown in two layers (organic layer contained secondary metabolites) and incubated overnight in a rotary shaker. The mixture was then centrifuged, and the supernatant was taken. The concentrated supernatant was further used for antimicrobial activity.

### 2.7. Antimicrobial Activity against ESBL-Producing *E. coli*

The ESBL-producing clinical isolate of *E. coli* was already confirmed by coauthors in the previous study [26]. Mueller–Hinton agar (MHA) plates were spread with test organisms (0.5 McFarland turbidity standard) using a sterile cotton swab. MHA plates were bored with 6 mm-diameter sterile cork borer, and 30 *μ*L ethyl acetate extract was loaded into wells. The plates were then incubated at 37°C for 24 h, and the zone of inhibition was measured [27]. To validate the experiment, negative control (ethyl acetate) and positive control (1 mg/mL neomycin) were also maintained, and each experiment was triplicated. We had also examined the antimicrobial activity of some isolates with *Staphylococcus aureus* (ATCC 25923), *E. coli* (ATCC 25922), and *K. pneumoniae* (ATCC 700603) by the perpendicular streaking method as primary screening.

## 3. Results

### 3.1. Characterization of *Streptomyces*

**Figure 1:**
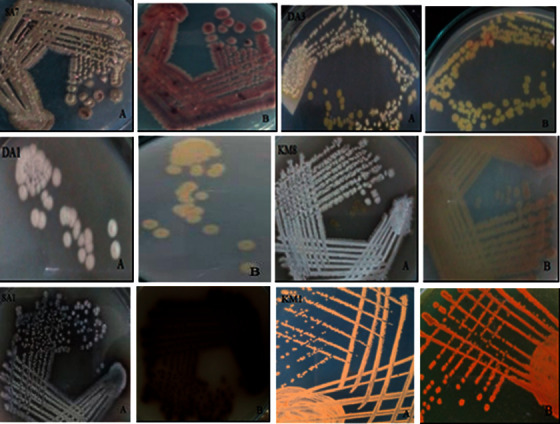
Colony morphology indicating aerial (a) and substrate (b) mycelia of isolated *Streptomyces* species.

**Figure 2:**
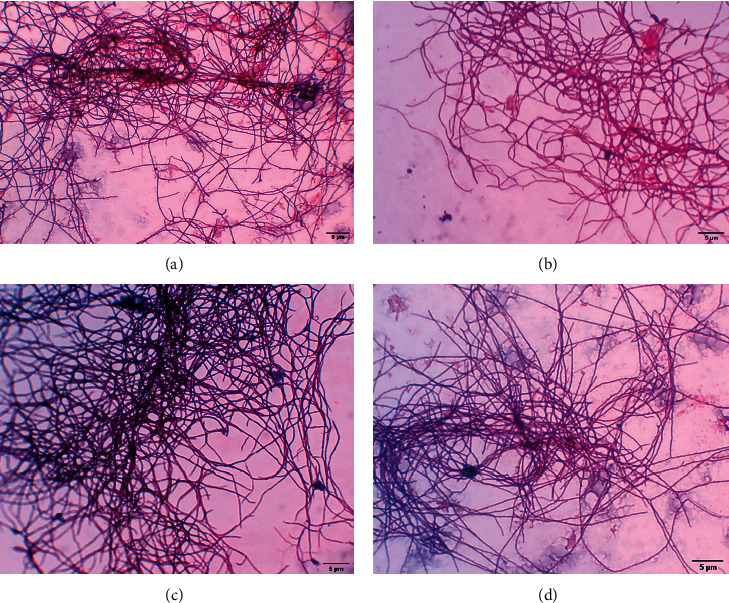
Gram staining of isolated actinomycetes under 100x magnification.

Soil samples collected from various environments varied in texture, and 18 *Streptomyces* isolates were successfully isolated (Table S1). Among them, no isolate was isolated from the soil of Rasuwa, two isolates from Thali, Dhangadi, Chitlang, Tapoban, and Taudaha soil and one isolate from each remaining eight types of soil. The isolates were identified as *Streptomyces* based on their mycelial and cellular morphology observed under a microscope and no other species such as *Nocardia* spp. and *Micromonospora* spp. were obtained. All these isolates attained maximum growth after seven days of incubation with colored sporulation ranging from dark grey, grey, dark brown, brownish, whitish, and yellowish-white, as indicated in Figure 1. The microscopic examination of actinomycete strains showed Gram-positive with hair-like mycelium, as shown in Figure 2. The physiological test showed that the isolates were nonmotile, unable to produce hydrogen sulfide, optimum growth between 28°C and 37°C, and able to tolerate up to 5% NaCl. In contrast, the sugar utilization test revealed that most of the strains under study were able to utilize glucose, arabinose, sucrose, xylose, and fructose but were not able to utilize mannitol, inositol, raffinose, and rhamnose.

### 3.2. Antibacterial Assays

**Figure 3:**
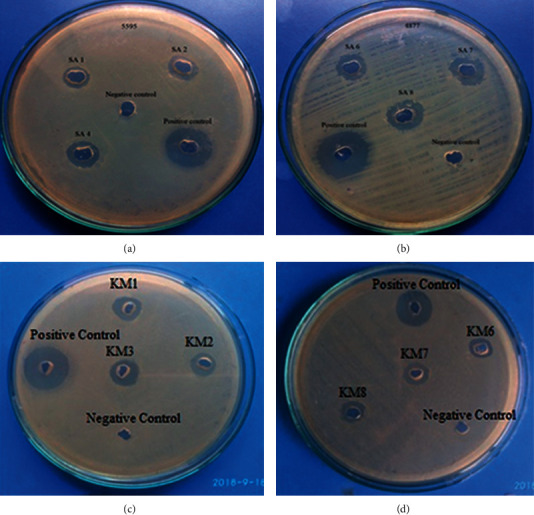
Antibacterial activity against ESBL-producing *E. coli* shown by crude extract of *Streptomyces* species.

The antibacterial activity of ethyl acetate extract (50 mg/mL) of isolates was evaluated against ESBL-producing clinical isolates of *E. coli* using well diffusion method. The results showed that crude extract exhibited a zone of inhibition ranging from 10 to 13 mm (size of wells: 6 mm) against ESBL-producing *E. coli* as compared to positive control neomycin with 19 mm zone of inhibition, as shown in Figure 3 and Supplementary Materials, Table S2. Some pure isolates also showed antimicrobial activity against *S. aureus* (ATCC 25923), *E. coli* (ATCC 25922), *K. pneumoniae* (ATCC 700603), and *Salmonella typhimurium* (ATCC 14028) (data not included in this report).

### 3.3. 16S rRNA Amplification

The PCR amplification of 16S rRNA using a set of universal primers 27 F and 1492 R resulted in ∼1.5 kb product as compared to 1 kb ladder (New England Biolabs) for all isolates. The amplified products were then purified with DNA Clean and Concentrator™-5 (Catalog no. D4003), Zymo Research (USA), following the manufacturer's instructions, and the DNA sequencing using universal primers was performed in Macrogen Inc., South Korea.

### 3.4. Molecular Characterization

The proximity of the 16S rRNA gene of the isolates was compared with the sequences available in public databases (GenBank, EMBL, and DDBJ) using the Nucleotide Basic Local Alignment Search Tool—BLASTN version 2.2.29 [28] Sequence homology was compared with 16S rRNA gene sequences available in the database using the FASTA algorithm. The 16S rRNA gene sequences of other *Streptomyces*, representing the type strains of *Streptomyces* species, were retrieved from the GenBank. The sequence was aligned using Clustal W ver. 2.01, and the phylogenetic tree was constructed using MEGA ver. 6 by the neighbor-joining method (Figure S1). The assigned GenBank DNA sequences DA-1, DA-2, DA-3, DA-4, KM-1, KM-2, KM-3, KM-5, KM-6, KM-8, SA1, SA2, SA3, SA4, SA4, SA5, SA6, SA7, and SA8. The accession numbers are mentioned in Table 1, and phylogenetic analysis revealed that 18 isolates shared some evolutionary similarities (Supplementary Materials, Figure S1).

### 3.5. Genome-Wide Analysis of 16S rRNA

The multiple copies of 16S rRNA are found in 27 *Streptomyces* species in their whole-genome sequences. In 7 species, DNA sequences in multiple copies of 16S rRNA are highly conserved, but in 20 others, there is little variation and per-site gaps in the aligned predicted by multiple sequences. *Streptomyces violaceusniger* Tu 4113 has only one 16S rRNA in the genome. The numerous copy of 16S rRNA present in the assembled genome sequencing of *Streptomyce*s is given in Supplementary Materials, Table S3. Our analysis revealed that DNA sequencing of a single copy of 16S rRNA in the genome could be sufficient for the identification of genus *Streptomyces* from class actinomycetes.

## 4. Discussion

The present study outlines molecular phylogeny, and antimicrobial activity of *Streptomyces* isolated from Nepalese soil. Till now, more than 7,000 compounds produced by *Streptomyces* species have been identified [6]. Continuous screening of *Streptomyces* will lead to the discovery of numbers of new compounds with diverse applications [29]. Thus, the reported 18 *Streptomyces* species from different environmental niches of Nepal would be beneficial for future antibiotics discovery program.

In this study, the ethyl acetate extracts showed antimicrobial activity against ESBL-producing *E. coli.* Previously, Ramachandran et al. have also suggested that ethyl acetate extract of actinomycetes has antibacterial activity against ESBL-producing *E. coli* [30], and this solvent is considered better for the extraction of active metabolites from actinomycetes [31]. In several reports, secondary metabolites from actinomycetes have shown the higher inhibition against Gram-negative bacteria; among them, *E. coli* is more susceptible. However, due to the presence of double-membrane barrier and transmembrane efflux, Gram-negative bacteria are more resistant to antimicrobial compounds as compared to Gram-positive bacteria [32]. Hence, actinomycetes that produce secondary metabolites against Gram-negative might play a significant role against antibiotic-resistant bacteria, such as ESBL-producing *E. coli.* Previous studies suggested that ethyl acetate extract of actinomycetes, isolated from Nepalese soil of different altitudes, can produce secondary metabolites, which showed antimicrobial activity against different ATCC strains [33–36].

To our knowledge, the antimicrobial activity against ESBL-producing *E. coli* using secondary metabolites of *Streptomyces* and their molecular characterization have not been reported earlier from Nepal.

From the analysis of partial 16S rRNA sequences deposited in GenBank (Table 1), it was revealed that all isolates belong to a species of the genus *Streptomyces* with 80–99% similarity with the 16S rRNA sequence of its closely related species. Bacterial genetic marker, 16S rRNA, often exists as a multigene family or operons in chromosomal DNA. The function of this gene over time has not been changed, and the size of this gene is large enough for the informatics purposes [37]. The complete gene sequence of 16S rRNA consists of approximately 1,500 bp with significantly conserved regions and nine variable regions that allow broad taxonomic spectrum and taxonomic discrimination, respectively. However, there is some discrepancy regarding the molecular identification of bacterial species based on 16S rRNA. Guo et al. have claimed that sequencing of 16S rRNA is not useful for closely related strains and only limited to the distinction of slightly related *Streptomycetes* [38].

Some studies have reported that multiple copies of 16S rRNA are limited and have less probability of affecting the phylogenetic analysis of the species because, in most cases, the sequence of these multiple copies is entirely or nearly identical [39–41]. However, others have shown that with an increase in the number of multiple copies of 16S rRNA, the variation in sequence also increases [42], and the 16S rRNA sequence is not enough criteria to assure species identity [43].

*In silico* analysis of 16S rRNA of 174 actinobacteria showed the phylum contains 3.402 ± 1.720 average copies of 16S rRNA, of which 1.569 ± 0.869 copies are not 100% similar to other copies. The results infer that the average maximum variation is 1.121 ± 2.400, along with an average minimum similarity of 99.927 ± 0.158 based on multiple copies of 16S rRNA analysis [44]. Ten *Streptomyces* strains isolated from three different lichens were found to have similar 16S rRNA gene sequences [45, 46]. The analysis of multiple copies of 16S rRNA in different *Nocardia* strains suggests that only BLAST analysis could not confirm the species present in the isolates [47]. Two copies of 16S rRNA genes contained in actinomycete, *Thermobispora bispora*, differ in nucleotides sequence at 98 positions (6.4% of complete nucleotides sequence) having six regions of insertion and deletions [48]. A study suggested transitional and transversional substitutions as two distinct mechanisms for the occurrence of multiple copies of 16S rRNA in 33 *Streptomyces* strains (6.9%) out of 475 isolates [49]. Thus, the sequencing of multiple copies of 16S rRNA seems to be not useful for the identification of species.

## 5. Conclusion

The present study demonstrates that *Streptomyces* species isolated from different altitudes of Nepal showed potential antibacterial activity against ESBL-producing *E. coli*. This report demands further study on *Streptomyces* spp. of Nepal to uncover novel secondary metabolites, which could be an ideal option for the treatment of infections caused by drug-resistant pathogens. We also suggest that the molecular sequencing of multiple copies of the 16S rRNA is not necessary for species identification of *Streptomycetes*, rather fatty acid methyl ester (FAME) analysis, profiling of metabolites, and DNA-DNA hybridization are required for this purpose.

## Figures and Tables

**Table 1:** 16S rRNA sequence analysis of *Streptomyces* species and potent secondary metabolites.

Sample no.	Accession number	Match in the databases		
		Species	Identity (%)	Identifier
SA1	LC427859	*Streptomyces* spp. ZG731	96	GQ985455.1
		*Streptomyces coelicoflavus* strain HQA809	93	KT758401.2
SA2	LC425654	*Streptomyces* spp. BCG69	80	KF956734.1
		*Streptomyces* spp. 219839	80	HQ992728.1
SA3	LC427860	*Streptomyces* spp. strain D3	97	KX762051.1
		*Streptomyces lividans* strain jx-02	97	KC898819.1
SA4	LC427861	*Streptomyces* spp. strain D3	98	KX762051.1
		*Streptomyces lividans* strain YLA0	98	KT362142.1
SA5	LC427862	*Streptomyces* spp. CMU-AC2	96	LC073311.1
		*Streptomyces* spp. CMU-AB225	96	LC073310.1
SA6	LC427863	*Streptomyces* spp. JSM 147611	96	KR817740.1
		*Streptomyces violascens* strain G8A-22	96	HQ238389.1
SA7	LC427864	*Streptomyces* spp. DHS C014	97	KP986577.1
		*Streptomyces tumenensis*	97	AM180560.1
SA8	LC427865	Uncultured *Streptomyces* spp. clone T1S-05	82	GQ369231.1
		*Streptomyces ryensis* strain zw24	83	MH337934.1
KM1	MT463712	*Streptomyces* spp. 34005	98	GU263848.1
		*Streptomyces* strain P14S1	98	KX673842.1
KM2	MT463715	*Streptomyces globisporus* subsp*. globisporus* isolate XSD*-*114	85.1	EU273549.1
		*Streptomyces cavourensis* strain TSM 11	85	MK789724.1
KM3	MT463732	*Streptomyces pratensis* strain EA5	93.2	KU973961.1
		*Streptomyces* spp. QLS12	93.5	KU973961.1
KM5	MT464457	*Streptomyces coelicoflavus* strain 3-2	98.3	KJ571034.1
		*Streptomyces coelicoflavus* strain HQA020	98.1	KT758352.1
KM6	MT464460	*Streptomyces* spp. 193322	96.2	KU982617.1
		*Streptomyces parvus* strain T23	96.2	KU317906.1
KM8	MT464462	*Streptomyces canus* strain IMCC 34906	97.4	MK138629.1
		*Streptomyces* spp. strain ST228	97.4	KX906839.1
DA1	MT441544	*Streptomyces globisporus* strain AHS10	99	KU981096.1
		*Streptomyces violascens* strain EA27	98.2	KU973982.1
DA2	MT459245	*Streptomyces naganishii* NRRLB-1816	98.4	NR_043831.1
		*Streptomyces* spp. FXJ1.447	98.4	KP126357.1
DA3	MT459275	*Streptomyces pratensis* 190525	97.1	KU973967.1
		*Streptomyces* spp. strain WA22-1-2	97	KY206810.1
DA4	MT463685	*Streptomyces* spp. strain HN25	98.7	MF397913.1
		*Streptomyces diastaticus* subsp. *ardesiacus* strain AR-39	98.7	KX055839.1

## Data Availability

DNA accession numbers are available on GenBank.

## Supplementary Materials

Supplementary Materials
*Supplementary Figure S1*. Neighbor-joining phylogenetic tree of 16S rRNA from 18 isolates. Horizontal branch lengths proportional to the estimated number of nucleotide substitutions, and bootstrap probabilities (as percentages), as determined for 1000 resamplings, are given *above* or *beside* the internal branches. The bar in the lower-left corner indicates 0.2 amino acid substitutions per amino acid for the branch length. *Supplementary Table S1*. Cultural features of *Streptomyces* species isolated from different parts of Nepal. *Supplementary Table S2*. Antimicrobial activity of some *Streptomyces* species against ESBL-producing *E. coli. Supplementary Table S3*. Multiple copy numbers of 16S rRNA present in the assembled genome sequencing of *Streptomyces* given in the website (JGI IMG Integrated Microbial Genomes and Microbiomes, 2019).
