# Supplementary material for: Molecular Identification and Antimicrobial Potential of Streptomyces Species from Nepalese Soil
Source: Int J Microbiol. 2020 Aug 27;2020:8817467. doi: 10.1155/2020/8817467 (PMC7474392; doi:10.1155/2020/8817467)
Supplement: Supplementary Materials — Supplementary Figure S1. Neighbor-joining phylogenetic tree of 16S rRNA from 18 isolates. Horizontal branch lengths proportional to the estimated number of nucleotide substitutions, and bootstrap probabilities (as percentages), as determined for 1000 resamplings, are given above or beside the internal branches. The bar in the lower-left corner indicates 0.2 amino acid substitutions per amino acid for the branch length. Supplementary Table S1. Cultural features of Streptomyces species isolated from different parts of Nepal. Supplementary Table S2. Antimicrobial activity of some Streptomyces species against ESBL-producing E. coli. Supplementary Table S3. Multiple copy numbers of 16S rRNA present in the assembled genome sequencing of Streptomyces given in the website (JGI IMG Integrated Microbial Genomes and Microbiomes, 2019). [file 8817467.f1.docx]

**Molecular Identification and Antimicrobial Potential of *Streptomyces* species from Nepalese soil**

### Karan Khadayat^1,2^, Dawa Dindu Sherpa^2^*^#^*, Krishna Prakash Malla^2^*^#^*, Sunil Shrestha^2^*^#^*, Nabin Rana^2^, Bishnu P Marasini^2^, Santosh Khanal^3^, Binod Rayamajhee^3^, Bibek Raj Bhattarai^1^ and Niranjan Parajuli^1*^

^1^Central Department of Chemistry, Tribhuvan University, Kirtipur, Kathmandu, Nepal

^2^Department of Biotechnology, National College, Tribhuvan University, Naya Bazar, Kathmandu, Nepal

^3^Department of Microbiology, National College, Tribhuvan University, Naya Bazar, Kathmandu, Nepal


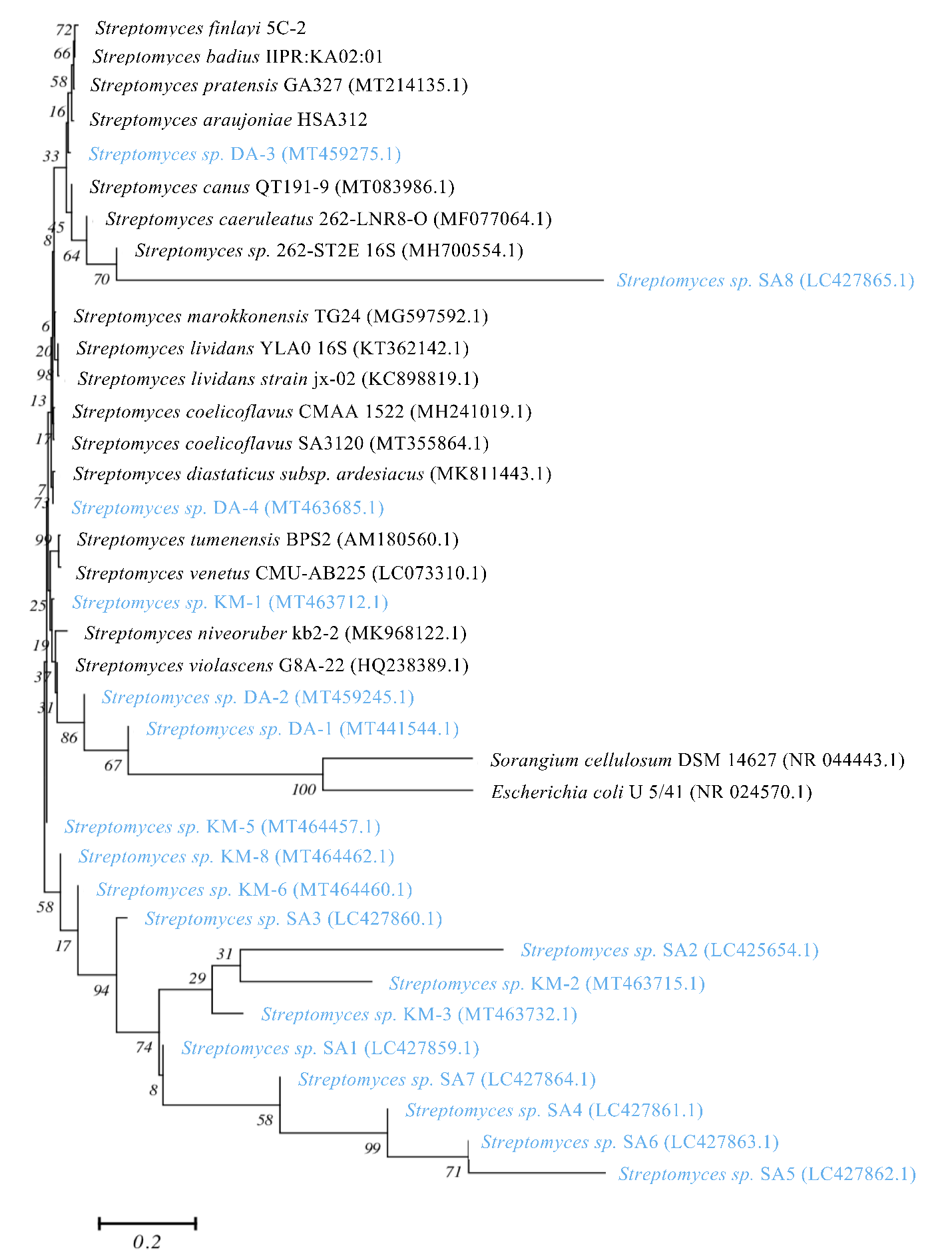


**Fig. S1** Neighbor-joining phylogenetic tree of 16S rRNA from 18 isolates. Horizontal branch lengths proportional to the estimated number of nucleotide substitutions, and bootstrap probabilities (as percentages), as determined for 1000 resamplings, are given *above* or *beside* the internal branches. The bar in the lower-left corner indicates 0.2 amino acid substitutions per amino acid for the branch length.

**Table S1.** Cultural features of *Streptomyces* species isolated from different parts of Nepal

| **Sample No.** | **Location** | **Aerial**  **mycelium** | **Substrate**  **mycelium** | **Latitude and Longitude** | **Height above**  **Sea level** |
| --- | --- | --- | --- | --- | --- |
| SA1 | Ratnanagar, Chitwan | White | Red | 27°37'2.4"N and 84°30'42.24"E | 196.66 m |
| SA2 | Thali, Kathmandu | Dark grey | Grey | 27° 43' 37.42" N and 85° 24' 12.68" E | 1386 m |
| SA3 | Godawari, Lalitpur | Whitish grey | Grey | 27°36'5.63"N and 85°21'55.07"E | 1439.16 m |
| SA4 | Thali, Kathmandu | White | Brown | 27° 43' 37.42" N and 85° 24' 12.68" E | 1368 m |
| SA5 | Dhangadi, Kailali | Yellow | Yellow | 28° 41' 6.88'' N and 80° 37' 17.73'' E | 180.91m |
| SA6 | Chitlang, Thaha | Yellow | White | 27°38'31.44"N and 85°10'44.54"E | 1819m |
| SA7 | Tapoban, Darchula | Grey | Pink | 29° 45' 36"N and80° 47' 24"E | 1709m |
| SA8 | Taudaha, Kirtipur | White | Pink | 27°38'56.04"N and 85°16'56.18"E | 1291 m |
| KM1 | Dhangadhi, Kailali | Chremy white | Orange | 28° 41' 6.88'' N and 80° 37' 17.73'' E | 180.91m |
| KM2 | Bhimdatta, Kanchanpur | White | Pink | 28°59'14.21"N and 80°9'54.67"E | 228.79m |
| KM3 | Taudaha, Kirtipur | Grey | Pink | 27°38'56.04"N and 85°16'56.18"E | 1291 m |
| KM5 | Jugal, Sindhupalchowk | White | Yellow | 27°50'11.31"N and 85°46'26.87"E | 866m |
| KM6 | Chitlang, Thaha | Grey | White | 27°38'31.44"N and 85°10'44.54"E | 1819m |
| KM8 | White Gumba | White | Brown | 27°43'10.86"N and 85°17'12.16"E | 1315.02m |
| DA1 | Tatopani, Sindhupalchowk | White | White | 27°58'48"N and 85°55'48"E | 3135.47m |
| DA2 | Rajbiraj, Saptari | White | Brown | 26°32'31.01"N and 86°45'24.2"E | 86.65 m |
| DA3 | Muktinath, Mustang | Yellow | Yellow | 28°48'6.3"N and 83°51'28.46"E | 4026 m |
| DA4 | Tapoban, Darchula | White | Pink | 29° 45' 36"N and80° 47' 24"E | 1709 m |

**Table S2.** Antimicrobial activity of some *Streptomyces* species against

ESBL producing *E. coli*

| S.No. | **Sample Number** | **Diameter of Zone of inhibition (mm)** |
| --- | --- | --- |
| 1 | SA1 | 10 |
| 2 | SA2 | 12 |
| 3 | SA4 | 13 |
| 4 | SA5 | 12 |
| 5 | SA6 | 11 |
| 6 | SA7 | 13 |
| 7 | SA8 | 13 |
| 8 | KM1 | 12 |
| 9 | KM2 | 10 |
| 10 | KM3 | 13 |
| 11 | KM6 | 11 |
| 12 | KM8 | 11 |
| NC | Ethyl acetate | - |
| PC | Neomycin (1mg/mL) | 19 |

**Table S3.** Multiple copy numbers of 16S rRNA present in the assembled genome sequencing of Streptomyces given in the website(JGI IMG Integrated Microbial Genomes & Microbiomes, 2019)

Where, NC=Negative control; PC= Positive control

| **Genome** | **Size** | **Gene count** | **16S rRNA Count** | **Variation in sequence (Gaps and mutation) *** |
| --- | --- | --- | --- | --- |
| *Streptomycesalbidoflavus* SM254 | 7170504 | 6307 | 7 | The alignments varied at site 1250 with a gap at site 1. |
| *Streptomycesalbulus* CK-15 | 9336218 | 8203 | 7 | The alignments varied only on one site 80 with a gap at site 1. |
| *Streptomyces* spp. fd1-xmd | 7929999 | 7164 | 7 | The alignments varied only on one site 197. |
| *Streptomycesgilvosporeus* F607 | 8482298 | 7598 | 6 | Only one variation found at site position 80. |
| *Streptomycesagglomeratus* 5-1-3 | 9595404 | 8949 | 7 | The alignments varied by a gap at site 1. |
| *Streptomycesdengpaensis*XZHG99 | 8710171 | 7982 | 6 | The alignments varied only at site 1249. |
| *Streptomyces hygroscopicuslimoneus* KCTC 1717 | 10537932 | 9395 | 6 | The alignment varied at sites 1234, 1016, 375, and 196 with a gap at site 1 |
| *Streptomyces* spp. S063 | 7614683 | 7035 | 6 | The alignment varied at site 89 with three gaps at sites 1006, 1312, and 1436. |
| *Streptomyceslunaelactis* MM109 | 8570191 | 7762 | 6 | The alignment varied at sites 940, 939, 161, 160, and 1 along with gaps at sites 1490, 1489, 1488, 1447, 1194, 1150, 1129, 1005, 1004, 974, 973, 907, 851, 850, 773, 748, 747, 656, 643, 602, 590, 589, 588, 587, 581, 580, 579, 516, 441, 354, 353, 352, 342, 280, 206, 165, 162, 161, 160, 130 |
| *Streptomycescattleya* NRRL 8057 | 8092553 | 7576 | 6 | The alignments varied at sites 1236, 990, 989, 986, 985, 980, 979, 977, 976, 975, 173, and 171. |
| *Streptomycesclavuligerus* F613-1 | 7590758 | 6302 | 6 | The alignments varied at site 1 with four gaps. |
| *Streptomyceslividans* TK24 | 8345283 | 7510 | 6 | The alignments varied at sites 988, 306, 183, and 1 with two gaps. |
| *Streptomycesqaidamensis* S10 | 9083372 | 8187 | 6 | The alignments varied at sites 999, 998, 997, 994, 987, 986, 985, 984, and 989. |
| *Streptomycesvenezuelae* NRRL B-65442 | 8380320 | 7624 | 7 | The alignments varied at sites 6 and 194. |
| *Streptomycesparvulus* 2297 | 7766531 | 7007 | 6 | The alignment varied at sites 960, 958, 955. 952, 937, 931, 886, 885, 883, 866, 848, 842, 837, 816, 814, 810, 807, 679, 674, 672, 667, 664, 653, 646, 570, and 197 along with two gaps at sites 5 and 6. |
| *Streptomycesautolyticus* CGMCC0516 | 10184660 | 8354 | 6 | The alignments varied at sites 149, 1000, 1001, 997, 996, 991, 990, 987, 986, 176, 200, and 176 along with a gap at site 150. |
| *Streptomycespurpureus* KA281, ATCC 21405 | 7458334 | 6892 | 7 | The alignments varied at sites 156, 583, and from 1-37 by 4 and 37 gaps.  One of the sequences has only 1-37 sites. |
| *Streptomycesalbidoflavus* J1074 | 6841649 | 5919 | 7 | The alignment varied at site 1105. |
| *Streptomycesmalaysiensis* DSM 4137 | 10744231 | 8779 | 6 | The alignments varied at sites 176, 265, 979, 986, 987, 990, 991, 996, 997, 1000, 1001, 1096, 1341, 1342, and 1441. |
| *Streptomyceshygroscopicusjinggangensis* TL01 | 10077952 | 8964 | 6 | The alignments varied at sites 176, 253, and 366. |
| *Streptomycesalbus* DSM 41398 | 8384669 | 6872 | 6 | All sequences are highly aligned without any variations and gaps |
| *Streptomycesalbus* BK3-25 | 8308430 | 6823 | 6 | All sequences are highly aligned without any variations and gaps |
| *Streptomycessilaceus* ACCC40021 | 8625867 | 7549 | 6 | All sequences are highly aligned without any variations and gaps |
| *Streptomycesglobisporus* C-1027 | 7783599 | 7031 | 6 | All sequences are highly aligned without any variations and gaps |
| *Streptomycesavermitilis* MA-4680 | 9119895 | 7792 | 6 | All sequences are highly aligned without any variations and gaps |
| *Streptomycesgriseusgriseus* NBRC 13350 | 8545929 | 7222 | 6 | All sequences are highly aligned without any variations and gaps |
| *Streptomycesviolaceusniger* Tu 4113 | 10988130 | 9557 | 1 | Only one copy |

**Photograph 1**

**Photograph 2**

**Photograph 3**
